# Case of Eversion and Distortion of the Lower Eyelid, Treated by a Plastic Operation

**Published:** 1849-01

**Authors:** W. W. Durham

**Affiliations:** Georgia


*Case of Eversion and distortion of the lower Eyelid, treated by a Plastic Operation.* By W. W. Durham, M. D., of Georgia. Reported by A. A. Bell, Student of Medicine.

The subject of this report, Mr. T. J. M., a young man aged 19, had the misfortune while quite young to fall from a waggon and be caught beneath its wheels, which passed partially over his body, obliquely across his face in the direction of the inner canthus of the eye, ranging with the cheek, producing a lacerated wound, in their whole trace, upon the face, extending upon the forehead, and it was thought fractured the frontal bone. The result of the injury was a considerable distortion of the lower eyelid, at the same time everting it. The lid was torn loose at its inner angle which was, by the contraction of the cicatrices, carried down upon the cheek fully an inch. The effect of this derangement of the parts was such as slightly to protrude the eyeball and to push it below the plane of its fellow. The upper eyelid, by being drawn upon by the contraction of the cicatrices, is made to cover a larger portion of the globe of the eye than natural. Along with the displacement, I would observe that the punctum lachrymale being carried down with the internal canthus, was rendered entirely incapable of performing the important office of removing the tears, and possibly the canal has been altogether obliterated, consequently a state of epiphora existed with the injury. By the long exposure of the conjunctiva to the action of the atmosphere, it has become quite thickened, presenting constantly the red appearance of vascular excitement. The cicatrix below the eye which acted most prominently in drawing the lid down, had formed through the medium of the cellular tissue strong adhesion to the periosteum. Thus exposing the eye and its tender lining to the contact of foreign bodies, and the impressions of atmospheric changes.

In this uncomfortable situation, after the lapse of six or eight years, the young man concluded to submit to an operation for the purpose of having the deformity and exposed condition of the eye overcome as much as possible. Dr. Durham was consulted, and on May the 6th, it was decided that a plastic operation should be performed, in the manner described by Professor Pancoast in his work on "Operative Surgery," and there ascribed to Lallemand. This was done in the presence of several medical gentlemen. To fulfil the indications demanded, a section was made beneath the lid an inch or more in length. The integument was then dissected up, so as to allow the lid to be replaced as much as the condition of the surrounding parts would admit, leaving a considerable gap to be filled. To supply this breach a flap was raised from the cheek, after the manner laid down by the author in the work before mentioned, (fig. 3, plate lxxiii.) twisted upon itself and laid in the interspace made by the previous section and confined by a few interrupted sutures. The wound upon the cheek was brought together in the same way. To approximate the edges of the cut surfaces more closely, a few straps of adhesive plaster were applied. Thus managed, without any cumbersome dressing, and with the observance of a strict antiphlogistic regimen, adhesion readily took place without any untoward symptom occurring. The flap for a day or so exhibited but little signs of sensibility, from which state, however, it soon recovered. During the operation the patient was placed under the influence of *chloroform* with a happy effect.

In addition to the operation described, it was thought the inner canthus could be made to unite at its original place of attachment. This was attempted but did not succeed entirely as the flow of the tears interfered, frustrating the adhesive process, leaving the parts, however, in no worse condition than before.

The effect of the operation has been to overcome in a great measure the everted condition of the distorted lid, though not to the same extent it would, were it not for the hypertrophied state of the conjunctiva, which at the time it was thought prudent not to interfere with. Its removal would greatly add to the success of the operation. This was deferred to a subsequent period, and would have been done, had the patient not objected. The young man who is quite intelligent, considers himself much benefitted.

I am aware that there is no new feature developed in this case, and although the operation has not succeeded equal to the sanguine wishes of the operator, it tends to show how much is to be expected from plastic surgery, notwithstanding an English surgeon of note (Fergusson) "would not be very sanguine of the happy results." Much has already been accomplished in this branch of the science, and the multiplied cases arm the surgeon with renewed confidence in the application of his art.

